# Higher incidence of meibomian gland dysfunction in postmenopausal women with primary acquired nasolacrimal duct obstruction

**DOI:** 10.1007/s10792-024-03041-9

**Published:** 2024-02-13

**Authors:** Guoping Wang, Haili Jin, Yonghong Sheng, Feng Ji, Yin Liu, Linfeng Han, Xiaohu Wang, Xianjie Chen, He Ding, Jing Liu, Qingqing Fu

**Affiliations:** Department of Ophthalmology, Wuhu Eye Hospital, Wuhu, 241002 Anhui Province China

**Keywords:** Primary acquired nasolacrimal duct obstruction, Higher incidence, Postmenopausal women, Meibomian gland dysfunction

## Abstract

**Purpose:**

This study aimed to investigate the incidence of meibomian gland dysfunction (MGD) in postmenopausal women with primary acquired nasolacrimal duct obstruction (PANDO) and enables ophthalmologists to pay attention to ocular surface damage before surgery.

**Methods:**

165 postmenopausal women with PANDO and 115 postmenopausal women with a normal lacrimal drainage system were enrolled in this prospective study. Based on the results of lacrimal duct irrigation and age, the participants were further subdivided. The incidence of different severities of MGD in different groups was calculated and analyzed by the chi-squared test.

**Results:**

The incidence of MGD in the PANDO group was 81.21%, and in the control group, it was 46.96%, which was significantly higher in the presence of PANDO (*p* < 0.001). The incidence of severe MGD in the complete and incomplete PANDO groups was higher than that in the control group (all *p* < 0.05), and no significant differences were observed between the complete and incomplete PANDO groups. The incidence of moderate MGD was significantly higher in the complete PANDO group than in the control group (*p* < 0.001). When age was considered an independent variable, the results revealed a significant value for patients aged < 70 years (*p* < 0.001).

**Conclusions:**

Our study revealed a prodominantly high incidence of MGD in postmenopausal women with PANDO, especially in a complete PANDO or aged < 70 years. Ophthalmologists need to pay close attention to MGD in postmenopausal women with PANDO.

## Introduction

Primary acquired nasolacrimal duct obstruction (PANDO) is a common ophthalmology condition that primarily affects menopausal females [1]. Chronic inflammation and nasolacrimal duct (NLD) fibrosis are considered to be the pathogenesis of PANDO [1]. PANDO is prone to ocular surface damage due to delayed tear clearance and increased levels of inflammatory cytokines [2].

Previous research has found that nearly 20–27% of patients with PANDO experience dry eye postoperatively and have no better or worse quality of life [3, 4]. However, whether patients with PANDO already had dry eyes before surgery was unclear. Meibomian gland dysfunction (MGD) is the primary cause of evaporative dry eye and is characterized by meibomian gland (MG) orifice and terminal duct obstruction due to hyperkeratinization, impairing ocular surface homeostasis [5]. Primary ocular surface inflammatory disease has been shown in the literature to impair MG structure and function [6, 7].

Previous studies have shown that the concentrations of inflammatory cytokines in the tears of patients with PANDO were higher than those of healthy participants [2, 8]. As the MG orifice is exposed to the tear film, inflammatory cytokines can impair MG orifices and initiate MGD [5, 9]. However, the incidence of MGD in patients with PANDO is still unclear, and since postmenopausal women are the most susceptible population to PANDO and MGD, we compared the incidence of MGD in postmenopausal women with PANDO and healthy controls to investigate the incidence of MGD in postmenopausal women with PANDO and help ophthalmologists pay close attention to MG impairment in postmenopausal women with PANDO before surgery to choose the best treatment strategies for both conditions.

## Materials and methods

### Participants

In this prospective study, 165 eyes of 165 postmenopausal women with PANDO (PANDO group; mean age 62.43 ± 8.17 years) and 115 eyes of 115 postmenopausal women with a normal lacrimal drainage system (control group; mean age 61.21 ± 8.57 years) were enrolled in Wuhu Eye Hospital, between March 2021 and February 2023. The affected eye was selected as the study eye for the experimental group, and the more severely affected eye was preferred when both were affected. In the control group, the right eye of participants was selected.

Participants in the PANDO group were diagnosed with PANDO based on irrigation and dacryocystography. Based on the results of lacrimal duct irrigation, the PANDO group was subdivided into an incomplete PANDO group (partial reflux, partial pharyngeal) and a complete PANDO group (complete reflux). The control group comprised participants who visited the hospital to improve their vision due to cataracts and healthy volunteers. The patient had normal lacrimal drainage, confirmed by irrigation in the outpatient clinic. Premenopausal women, those with continuous eye drop use, systemic disorders, history of ocular trauma or surgery, long-term contact lens use, ocular inflammation, diabetes mellitus, Sjögren’s syndrome, or other treatments that impair tear film quality and stability were excluded.

### Examinations

**Fig. 1:**
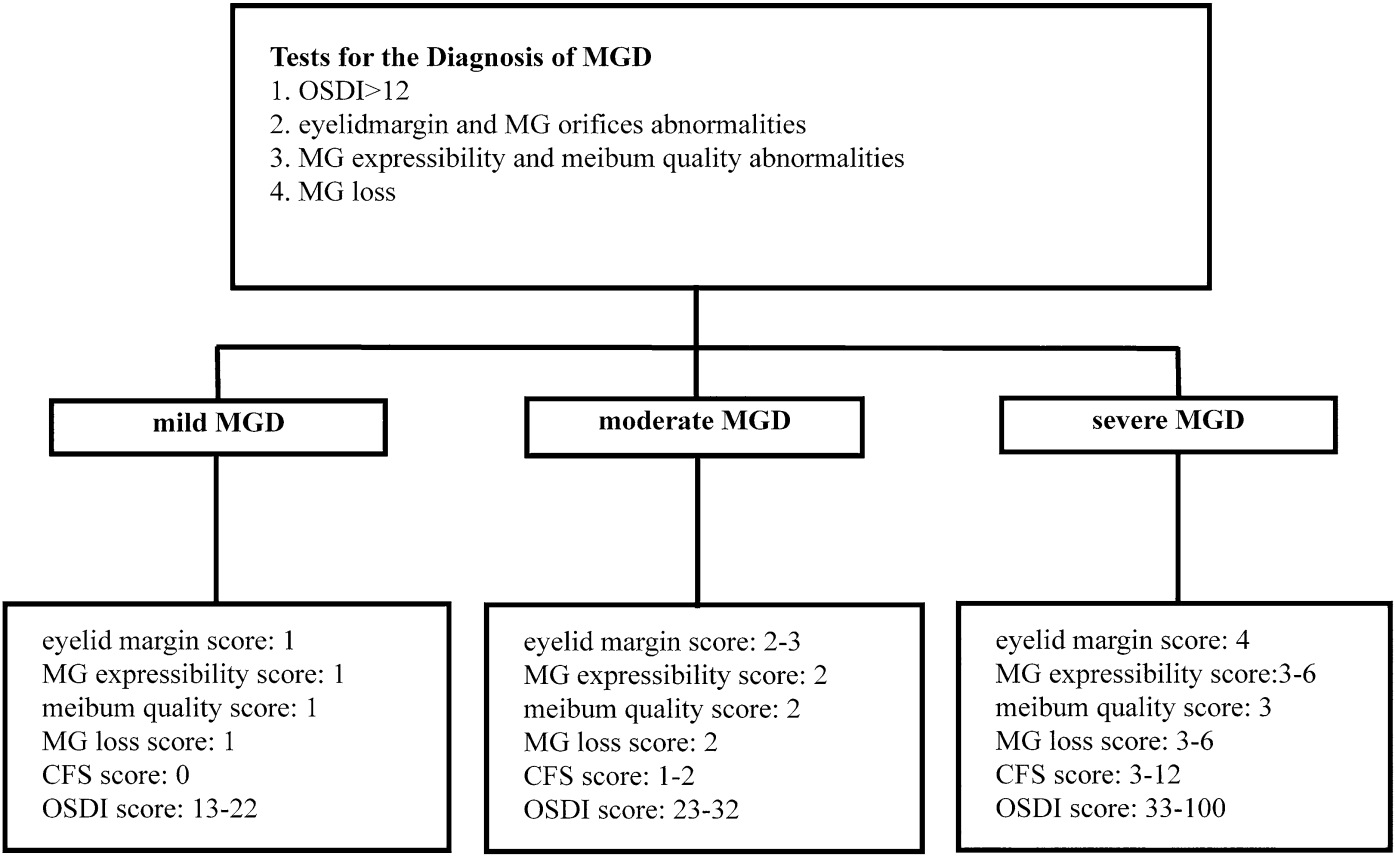
The diagnosis and severity levels of MGD. MGD meibomian gland dysfunction

Both participants and the professional operator were masked to the allocation. All measurements were conducted between 9 and 11 a.m. temperatures in the examination room ranged from 22 to 28 °C, and relative humidity levels were in the 40–50%. The ocular surface parameters were assessed sequentially. The ocular surface disease index (OSDI) questionnaire was used to assess ocular discomfort. The total score on the questionnaire ranges from 0 to 100, with a higher score indicating more severe ocular discomfort symptoms [10]. MG loss was examined using Keratograph 5 M (Oculus GmbH, Wetzlar, Germany). For each eyelid, MG loss was graded as grade 0 (no MG loss), grade 1 (MG loss < 1/3 of the total MGs), grade 2 (MG loss was 1/3–2/3 of the total MGs), and grade 3 (MG loss was > 2/3 of the total MGs) [11, 12]. The total MG loss score in the upper and lower eyelids was calculated. The four corneal zones (superior nasal, inferior nasal, superior temporal, and inferior temporal) were each given a score of 0 (no staining), 1 (1–30 dots of staining), 2 (staining between 1 and 3), or 3 (filaments, confluent stains, or ulcers). The corneal fluorescein staining (CFS) score was the total of these four scores [13]. The anterior/posterior shifting of the mucocutaneous junction, vascular engorgement, occluded MG orifices, and irregular eyelid margin on each eyelid was all assessed using slit-lamp diffused light and given a 0 (absent) or 1 (present) score [14]. The overall score ranged from 0 to 4. MG expressibility was evaluated by digitally pressing five MGs at the center of the upper eyelid [15]. The number of MGs from which meibum could be expressed was quantified on a scale of 0–3: 0 (all 5 MGs), 1 (3–4 MGs), 2 (1–2 MGs), and 3 (0 MGs). Meibum quality was evaluated from the center of the eight MGs in the upper and lower eyelids [16]. The meibum quality score ranged from 0–3 as follows: 0 (clear), 1 (cloudy), 2 (cloudy with debris), and 3 (inspissated). The diagnosis and severity levels of MGD were determined through assessment of ocular surface parameters [12]: eyelid margin, MG expressibility, meibum quality, MG loss, CFS, and OSDI (Fig. 1).

### Statistical analysis

The SPSS software package (version 22.0; SPSS Inc., Chicago, IL, USA) was used to perform statistical analyses. The incidence of MGD was compared using the chi-squared test. The normality of the data distribution was assessed using the Kolmogorov–Smirnov test. The Mann–Whitney *U* test was used to compare the ocular surface parameters between the two groups. Statistical significance was set at *p* < 0.05.

## Results

### Study participants’ clinical characteristics

The mean age and menopausal duration of the PANDO group were 62.43 ± 8.17 years and 12.31 ± 8.91 years, respectively. The mean age and menopausal duration of the control group were 61.21 ± 8.57 years and 10.89 ± 8.46 years, respectively. No significant differences were observed in age or menopausal duration between the PANDO and control groups (*p* = 0.384 and *p* = 0.363, respectively). The demographic characteristics of study participants are shown in Table 1.

**Table 1:** Demographic characteristics of study participants

	Age groups			Degree of obstructed duct	
	45–60	60–70	≥ 70	Incomplete	Complete
PANDO (*n* = 165)	89	38	38	67	98
Control (*n* = 115)	48	44	23		

*PANDO* primary acquired nasolacrimal duct obstruction

### Comparison of the ocular surface parameters and incidences of MGD in the PANDO and control groups

The total incidence of MGD in the PANDO group (81.21%) was significantly higher than that in the control group (46.96%, *p* < 0.001). Severe MGD was much more common in the PANDO group (46.06%) than in the control group (21.74%, *p* < 0.001). There were no discernible statistically significant variations in the frequency of other MGD severity groups. The OSDI score (33.33 [18.92, 53.04]) was considerably higher in the PANDO group than in the control group (13.89 [2.78, 25.00]) (*p* < 0.001). The MG loss score was higher in the PANDO group (3 [2, 4]) than in the control group (2 [2, 3]) (*p* = 0.004). The eyelid margin score was higher in the PANDO group (3 [2, 4]) than in the control group (2 [1, 3]), (< 0.001). There was no significant difference in the CFS score, MG expressibility score, or meibum quality score between the PANDO group and the control group (Table 2). The CFS score, meibum quality score, and MG expressibility score did not significantly vary between the PANDO and control groups (Table 2).

**Table 2:** Comparison of the ocular surface parameters and incidences of different severity of MGD in each group

	PANDO group (*n* = 165)	Control group (*n* = 115)	*p*
OSDI score	33.33[18.92,53.04]	13.89[2.78,25.00]	< 0.001^ǂ^
MG loss score	3[2, 4]	2[2, 3]	0.004^ǂ^
CFS score	1[0,1]	1[0,2]	0.280^ǂ^
Eyelid margin score	3[2, 4]	2[1, 3]	< 0.001^ǂ^
MG expressibility score	2[1, 2]	2[1, 2]	0.691^ǂ^
meibum quality score	3[2, 4]	3[2, 4]	0.695^ǂ^
Total MGD incidence	134 (81.21%)	54 (46.96%)	< 0.001*
Mild MGD incidence	7 (4.24%)	5 (4.35%)	0.966*
Moderate MGD incidence	51 (30.91%)	24 (20.87%)	0.062
Severe MGD incidence	76 (46.06%)	25 (21.74%)	< 0.001*

*PANDO* primary acquired nasolacrimal duct obstruction, *MGD* meibomian gland dysfunction, *OSDI* ocular surface disease index*, MG* meibomian gland*, CFS* corneal fluorescein staining

^ǂ^Kruskal–Wallis *H* test,, *Chi-squared test

### Comparison of incidences of MGD in the incomplete, complete PANDO, and control groups

The total incidence of MGD in the complete PANDO group (88.78%) and incomplete PANDO group (70.15%) was substantially greater than in the control group (46.96%, *p* < 0.001). The total incidence of MGD in the complete PANDO group (88.78%) was significantly higher than that in the incomplete PANDO group (70.15%, *p* < 0.001). The incidence of severe MGD in the complete PANDO group (47.96%) and the incomplete PANDO group (43.28%) was significantly higher than that in the control group (21.74%, *p* < 0.001); However, no significant difference was found in the incidence of severe MGD between the incomplete and complete PANDO groups. The complete PANDO group (47.96%) had a considerably greater incidence of moderate MGD than the control group (21.74%, *p* < 0.001); However, there was no discernible difference in the incidence of moderate MGD between the incomplete and complete PANDO groups or between the incomplete PANDO and control groups. There were no statistically significant differences in the incidence of mild MGD among the three groups (Table 3).

**Table 3:** Comparison of incidences of MGD in the incomplete, complete PANDO, and control groups

	Total MGD	Mild MGD	Moderate MGD	Severe MGD
Incomplete PANDO (*n* = 67)	47 (70.15%)	2 (2.99%)	16 (23.88%)	29 (43.28%)
Complete PANDO (*n* = 98)	87 (88.78%)	5 (5.10%)	35 (35.71%)	47(47.96%)
Control (*n* = 115)	54 (46.96%)	5 (4.35%)	24 (20.87%)	25 (21.74%)
χ^2^	42.30	0.459	6.325	17.76
*P* value	< 0.001	0.795	0.042	< 0.001

*PANDO* primary acquired nasolacrimal duct obstruction, *MGD* meibomian gland dysfunction;*chi-squared test*

### Comparison of incidences of MGD in each age group

When age was considered an independent variable, the incidences of MGD in patients with PANDO aged 45–60 years and 60–70 years were considerably greater than those in the control group. When the age was ≥ 70 years, there was no discernible difference in the incidence of MGD between the two groups (Table 4).

**Table 4:** Comparison of incidences of MGD in each age group

	45–60 years	60–70 years	≥ 70 years
PANDO	69 (77.53%)	34 (89.5%)	31 (81.6%)
Control	22 (44.9%)	17 (38.6%)	15 (65.2%)
χ^2^	14.982	20.302	2.068
*P*	< 0.001	< 0.001	0.150

*PANDO* primary acquired nasolacrimal duct obstruction; *chi-squared test*

## Discussion

Previous researches showed patients with PANDO experience dry eye postoperatively and may have preoperatively developed dry eye [5, 6]. However, typical dry eye symptoms in patients with PANDO complicated with MGD are frequently covered by epiphora, ophthalmologists tend to ignore dry eye in patients with PANDO, miss the best treatment strategy for both conditions. In this study, we determined that the incidence of MGD was considerably higher in postmenopausal women with PANDO than in postmenopausal women with a normal lacrimal drainage system and mostly showed a considerable increase in the prevalence of severe and moderate MGD.

Based on population-based studies, the incidence of MGD ranges from 3.5 to 69.3%, with a lower incidence in white populations and a higher incidence in Asian populations [17, 18]. In our study, about 54 (46.76%) of 115 postmenopausal women with normal lacrimal drainage systems met the MGD criteria. Our participant population included all Asian, suggesting considerable consistency with previously published findings regarding the incidence of MGD. Of the 165 postmenopausal women with PANDO, 134 (81.21%) met the MGD criteria. The percentage of postmenopausal women with MGD in the PANDO group (81.21%) was significantly higher than that in the control group (46.6%). Our findings suggest postmenopausal women with PANDO have a significantly high incidence of MGD.

When the NLD is blocked, severe inflammation and infection can occur, often with atypical flora, and fragments of the lacrimal sac can wash back into the tear film (the so-called volume sign) [19], increasing the risk of MGD. Several studies have reported that inflammation of the eyelid margin and palpebral conjunctiva is associated with changes in the structure and underlying function of the MG [6, 7, 20]. A similar mechanism might occur in the nasolacrimal drainage system. For higher incidence of MGD in postmenopausal women with PANDO, three mechanisms can be proposed. First, NLD hinders tear clearance and leads to the accumulation of inflammatory factors in the tear film, inducing inflammation of the MG orifice. Inflammation plays an important role in the pathogenesis of MGD [5]. Furthermore, owing to the delay in tear clearance, the exposure duration of the MG orifice is prolonged, resulting in a constant increase in inflammation and impairment of the MG. Second, because of the lack of removal of inflammatory cytokines and pathogenic microorganisms and their concentration by tear film evaporation, the poor environment is easily exploited by periocular commensal bacteria, which in turn proliferate and markedly exacerbate already significant chronic conjunctivitis. Subconjunctival inflammatory cells may directly damage the acina [21]. Bacteria may be key factors in the third pathway. According to previous reports, MGD correlates with changes in the types and quantities of microorganisms on the ocular surface [22]. Through bacterial lipid-degrading enzymes, microorganisms on the ocular surface mostly break down taribis lipids, resulting in changes in the lipid profile or in bacterial genes that promote the onset of inflammation and exacerbate diseases [23, 24]. The lacrimal passage communicates with the ocular surface and nasal cavity, and these microorganisms may affect the structure and function of the MGs.

In this study, we found significant differences in the incidence of total MGD between the complete and incomplete PANDO groups. However, no significant difference was observed in the incidence of different severity MGD between the patients in the complete and incomplete PANDO groups, suggesting no significant difference in the impact of the degree of NLD obstruction on the aggravation of MGD. On the one hand, in this study, the duration did not show a significant difference between the incomplete and complete PANDO groups. Therefore, the time of delayed tear clearance or damage to the ocular surface caused by harmful substances in the tears might be no significant difference. On the other hand, previous studies have reported that incomplete NLD increases blood flow resistance and tear stasis time [25], and the accumulated debris in the NLD enhances the malignant inflammatory cycle in the NLD mucosa [26, 27].

According to this study, the incidence of MGD was greater in the PANDO groups of people aged 45–60 years and 60–70 years compared to the control group. Patients with PANDO have a much higher incidence of MGD than those in their 50 s and 60 s. Therefore, when diagnosing and treating patients with PANDO, physicians should be aware of MG impairment in individuals aged between 50 and 60 years. Previous research has revealed that aging plays a significant role in the development of MGD [28] because of the decrease in meibocyte differentiation [29], MG atrophy [30], and impact on the major meibum lipids' composition ratio, which causes the emergence of subjective symptoms [31]. In this study, we discovered no discernible difference in the incidence of MGD between patients with PANDO and controls beyond the age of 70 years. These results suggest that when beyond 70 years old, aging may have a more important effect than PANDO on MGs.

However, this study had a limitation. We only observed postmenopausal women in this investigation. Therefore, we could not determine whether the incidence of MGD was exacerbated in males. Future studies will aim to include males to provide a comprehensive analysis of the impact of PANDO on ocular surface health.

## Conclusions

Our study showed that the incidence of MGD was significantly higher in postmenopausal women with PANDO. Ophthalmologists need to pay close attention to ocular surface damage in postmenopausal women with PANDO, especially in a complete PANDO or aged < 70 years. Further studies are recommended to determine the etiological relationship and whether early diagnosis and treatment of PANDO can reduce the incidence of MGD in patients with PANDO.

## Data Availability

The data sets used and analyzed during the current study are available from the corresponding author upon reasonable request.

## Abbreviations

NLD: Nasolacrimal duct
PANDO: Primary acquired nasolacrimal duct obstruction
MG: Meibomian gland
MGD: Meibomian gland dysfunction
OSDI: Ocular surface disease index questionnaire
CFS: Corneal fluorescein staining
